# Comparison between Tissue Adhesive Cyanoacrylate and Suture for Palatal Fibromucosa Healing: A Randomized Controlled Study

**DOI:** 10.3390/ma14227009

**Published:** 2021-11-19

**Authors:** Carlota Castro-Gaspar, Maria Victoria Olmedo-Gaya, Maria Nuria Romero-Olid, Maria Jesús Lisbona-Gonzalez, Marta Vallecillo-Rivas, Candela Reyes-Botella

**Affiliations:** 1 Department of Stomatology, Faculty of Dentistry, Campus de Cartuja s/n, University of Granada, 18071 Granada, Spain; carlotacastro@correo.ugr.es (C.C.-G.); mvolmedo@ugr.es (M.V.O.-G.); nromero@ugr.es (M.N.R.-O.); mjlisbona@icloud.com (M.J.L.-G.); mvallecillo@correo.ugr.es (M.V.-R.); 2 Medicina Clínica y Salud Pública PhD Programme, Faculty of Dentistry, University of Granada, 18071 Granada, Spain

**Keywords:** wound healing, guided periodontal tissue regeneration, cyanoacrylate, autografting, suture, bleeding

## Abstract

Cyanoacrylate tissue adhesive is proposed to promote soft tissue healing in oral surgery and minimize complications (pain, inflammation, and bleeding) associated with wound healing by secondary intention. The objective was to compare cyanoacrylate tissue adhesive (test group) with suture (control group) in terms of postoperative complications, operative time, and wound healing in the palatal donor area after harvesting a de-epithelialized gingival graft. A randomized controlled clinical trial was performed in 24 patients randomly assigned to one of two study groups. Data were gathered on wound bleeding, operative time, postoperative pain, inflammation, hyperesthesia, necrosis, and donor area healing time. Operative time was almost 50% shorter in the tissue adhesive cyanoacrylate group, a significant between-group difference (*p* = 0.003). Spontaneous bleeding in the donor area during the first 24 h was observed in 11.1% of the tissue adhesive cyanoacrylate group versus 88.9% of the suture group—a significant difference. No significant between-group difference was observed in postoperative pain, inflammation, or degree of healing over time. There were no cases of hyperesthesia or wound necrosis. Utilization of tissue adhesive cyanoacrylate rather than suture in palatal de-epithelialized gingival graft harvesting reduces postoperative bleeding during the first 24 h, as well as the operative time.

## 1. Introduction

Epithelial and epithelial-connective tissue grafts are considered the gold standard treatment for gingival recession owing to their biocompatibility and long-term stability [1]. The most frequent donor area is the palate, commonly using the technique described by Zuchelli et al. [2] to harvest a free graft (epithelial-connective tissue), which can then be de-epithelialized to give rise to a de-epithelialized connective or gingival graft (DGG). This is because the graft can be readily obtained at this site, even in patients with thin palatal mucosa, and there is a greater availability of the resulting tissue. The main drawback of this approach is the bloody bed left in the palatal area, which heals by secondary intention. This bed has been associated with postoperative pain and a risk of hemorrhage, inflammation, infection, and possible necrosis of the surgical wound [3,4,5]. Alongside suture, various hemostatic and wound healing agents have been used to accelerate the healing and reduce the prolonged bleeding and pain caused by the palatal wound, including absorbable synthetic collagen; absorbable gelatin sponges; oxidized regenerated cellulose; ferric subsulfate; and, more recently, cyanoacrylate cements and platelet-rich fibrin [6]. 

Cyanoacrylate adhesives have long been used in general surgery as an alternative to suture for surgical wound closure. They have been applied for multiple purposes in oral surgery, including periodontal dressing, sinus membrane perforation closure, bone fragment stabilization during fracture fixation, and peripheral nerve anastomosis closure [7]. They have also been proposed for wound closure by primary intention [8] and, more recently, for coating bloody surfaces healed by secondary intention [8,9,10]. Cyanoacrylate cements are formed by acrylic resins [11], and their mechanism of action is based on polymerizing monomers that create a layer to isolate the surgical area. The adhesive closes small capillaries, forms a protective barrier against trauma from food detritus or hygiene measures, blocks nociceptive nerve endings, and finally exerts a bacteriostatic effect [12,13]. The excellent hemostasis, fast adhesion to tissues, and bacteriostatic potential of cyanoacrylate tissue adhesives make them of major interest in surgery, and they can be more rapidly applied in comparison with conventional suture [14].

With this background, the main objective of this study was to compare the cyanoacrylate tissue adhesive versus suture. The null hypotheses were as follows: (i) there is no difference in bleeding between the cyanoacrylate and suture; (ii) there are no differences in operative time, postoperative complications, and time to wound healing in the palatal donor area.

## 2. Materials and Methods

### 2.1. Study Design and Patient Selection

A randomized controlled clinical trial with a parallel design was undertaken in patients requiring DGG harvesting from palatal fibromucosa to treat isolated gingival recession defects in mandibular or maxillary anterior teeth. Participants were treated at the Clinic of the Master’s Course of Granada University (Spain) between October 2018 and January 2020. The patients signed their informed consent to participation in the study, which was approved by the Ethics Committee of the University of Granada (number 870/CEIH/2020) and complied with the principles of the Helsinki declaration (2000 revision). This study was registered in the Australian New Zealand clinical trial registry (ANZCTR), number 382594, and followed the recommendations of the CONSORT 2010 statement for reporting randomized trials.

The sample size was estimated to obtain statistical power of 99% and a significance level of 99% to detect a reduction in bleeding of 40% in the treatment versus control group, considering a sample size ratio of control to experimental groups of 1.5. Study inclusion criteria were as follows: age between 18 and 60 years, low-moderate anesthetic risk (ASA I–II), no smoking habit or <10 cigarettes/day, and absence of active periodontal disease. Systemic diseases such as diabetes mellitus or bleeding syndromes were exclusion criteria. Participants were assigned to the test (cyanoacrylate) or control (suture) group using a computer-generated randomization sequence. This sequence was placed in sealed and opaque envelopes including the patient number and the randomization code, which was only revealed prior to surgery. Moreover, the examiners were previously recruited and trained to take an adequate measurement.

### 2.2. Surgical Protocol

Grafts were harvested by means of the epithelial-connective tissue graft technique described by Zucchelli et al. [2] for their subsequent de-epithelialization. Gauze soaked in saline solution was used to apply pressure on the donor surface for 5 min until the bleeding was controlled. Once the bleeding ceased, suture or cyanoacrylate tissue adhesive was applied. No additional hemostatic procedure was used.

The group assignation of each patient was communicated to the operator in a sealed envelope that was opened immediately after completing the graft extraction procedure. The wound was sutured with horizontal crossed mattress stitches (3/0 silk) (Arago^®^, Barcelona, Spain) in the control group or with cyanoacrylate adhesive (Periacryl^®^90, GluStitch Inc., Delta, Canada), following manufacturer’s instructions, in the test group. All patients were administered with amoxicillin (750 mg/8 h) (Clamoxyl^®^, Glaxosmithkline, Madrid, Spain) as an antibiotic prophylaxis from 24 h before until 6 days after the surgery. They were also prescribed 600 mg ibuprofen every 8 h for the first 4 days post-surgery.

After the intervention, all patients received a form for the evaluation of postoperative pain during the first 7 days post-surgery using a visual analogue scale (VAS) and to report any spontaneous bleeding of the surgical wound during this period.

### 2.3. Study Variables

The primary outcome of this study was spontaneous bleeding. Secondary outcomes were operative time, postoperative pain, inflammation degree, hyperesthesia, palatal mucosa necrosis, and healing time.

Data were gathered on the operative time used for the intervention (from the start of cyanoacrylate adhesive application or the picking up of the needle holder until the end of the corresponding palatal wound treatment); postoperative pain, evaluated on a VAS (0 = no pain to 10 = worst imaginable pain), preferably at the same time of day, every day for 7 days and at 14 days post-surgery; inflammation degree, measured on a verbal rating scale (VRS) (0 = no inflammation, 1 = mild inflammation, 2 = marked inflammation, and 3 = extreme inflammation) at 7, 14, and 21 days post-surgery; spontaneous bleeding (no/yes) during the first 7 days; presence of hyperesthesia, using a VRS (1 = none; 2 = mild, with no interference in normal life activities; and 3 = severe, with interference in normal life activities); palatal mucosa necrosis (no/yes); and healing time, up to the formation of the first epithelial layer as evaluated visually and by pressure with periodontal probe, and the time of restitutio ad integrum of the treated palatal fibromucosa. 

All patients attended four follow-up sessions (at 7, 14, and 21 days and 2 months) to evaluate healing outcomes and the presence/absence of hyperesthesia and necrosis. At the first follow-up (7 days), the completed pain/bleeding evaluation form was gathered from the patients, and the sutures were removed in control group patients.

### 2.4. Statistical Analysis

SPSS v 24.0 (SPSS Inc., Chicago, IL, USA) was used for the statistical analysis. In the descriptive analysis of the results for each group, contingency tables and bar charts were constructed for qualitative variables, and mean values with standard deviation (SD) and standard error of the mean, median, minimum, and maximum values and quartiles were calculated for quantitative variables. The normality of variable distribution was checked using the Shapiro–Wilk test.

The Mann–Whitney U test was used to compare quantitative variables (times, pain) and Fisher’s exact test (2 × 2 tables) or the chi-square test to compare qualitative variables (bleeding, inflammation, and healing) between groups. α = 0.05 was considered significant in all tests.

## 3. Results

The inclusion criteria for this study were met by 26 patients, assigning 14 to the control group (suture) and 12 to the test group (cyanoacrylate adhesive). One patient in the test group missed the follow-up sessions and another did not follow the medication protocol. The final study sample thus included 14 patients in the suture group (8 females) and 10 in the cyanoacrylate adhesive group (4 females).

**Figure 1:**
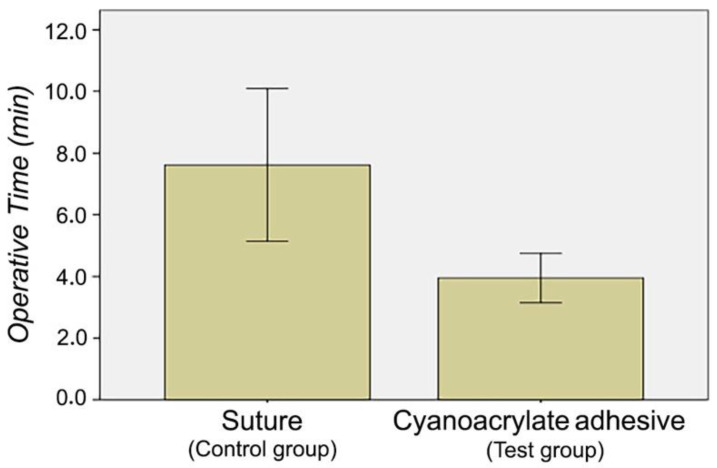
Comparison of operative time between suture and cyanoacrylate adhesive groups.

The mean ± SD operative time was 3.95 ± 1.27 min in the cyanoacrylate adhesive group versus 7.61 ± 4.64 min in the suture group—a statistically significant difference (*p* = 0.003) (Figure 1).

Pain was most intense at 24 h (4.21 ± 2.89 in the suture group and 3.20 ± 1.87 in the cyanoacrylate group) and then decreased, remaining almost constant during the first week. No statistically significant between-group difference in pain was observed at any time point (day 1, 2, 3, 4, 5, 6, 7, or 14) (Table 1).

No statistically significant between-group differences in palatal fibromucosal inflammation were found at any time point. The inflammation was marked in 71% of the suture group versus 50% of the cyanoacrylate tissue adhesive group at 7 days. It was mild or absent in 90% of the cyanoacrylate tissue adhesive group versus 71.4% in the suture group at 14 days and there was no inflammation in 70% versus 57.1 %, respectively, at 21 days (Table 2).

**Figure 2:**
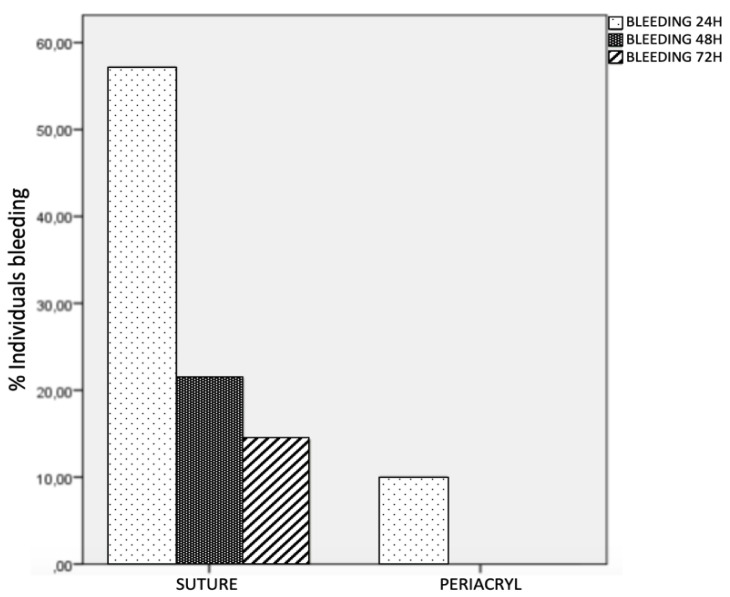
Bleeding at 24, 48, and 72 h.

Significant between-group differences (*p* = 0.033) were found in the presence of spontaneous bleeding during the first 24 h, which was reported by 57.1% of the suture group versus 10% of the cyanoacrylate tissue adhesive group. No significant difference was observed at 48 or 72 h. From day 3 onwards, no patient reported any spontaneous bleeding (Table 3 and Figure 2).

No statistically significant between-group difference was found in healing time (*p* = 0.665). A thin epithelial layer was observed in all patients (in both groups) at day 21, and restitutio ad integrum was confirmed in all patients at 2 months (Table 4).

There were no cases of palatal necrosis or hyperesthesia in either group.

The first null hypothesis was not met: (i) there is no difference in bleeding between the cyanoacrylate and suture. The second null hypothesis was not completely met: (ii) there are no differences in operative time, postoperative complications, and time to wound healing in the palatal donor area.

## 4. Discussion

In this pilot trial on the harvesting of palatal DGGs to treat gingival recession defects, lower pain outcomes and less frequent bleeding in the first 24 h post-surgery were achieved with the application of cyanoacrylate tissue adhesive than with suture, with no significant between-group differences in postoperative pain, inflammation, or healing time.

The palatal connective tissue graft technique described by Edel [15] and developed by Langer and Langer [16] is considered the gold standard approach in mucogingival surgery for gingival recession defects and for keratinized gingival width augmentation. The main drawback of this procedure is the postoperative morbidity caused by the additional surgical wound in the palate. Connective tissue extraction techniques have been developed to reduce this morbidity (Liu et al.) [17], such as closure by primary intention (Zuhr et al.) [18], including the window technique, single-incision technique, and inverted L technique, among others. The technique for free gingival graft harvesting described by Zucchelli and coworkers is one of the most frequently applied and considered the approach of choice [2,19,20]. As noted above, this is because it is relatively easy technique and does not require a palatal mucosa width of at least 3–4 mm. It leaves a surgical wound that heals by secondary intention and has not been associated with increased postoperative morbidity in comparison with other connective tissue extraction techniques [2]. However, patients generally experience pain for a few days after the surgery, especially during the first 48 h [3,21], and various procedures have been proposed to address this complication, including the use of butyl-cyanoacrylate adhesive with platelet-rich fibrin or alone [6].

Tissue adhesives, such as cyanoacrylate, have also been applied in oral surgery to achieve good surgical wound healing and take advantage of their hemostatic and antimicrobial properties. Nevins et al. [22] used this adhesive as an alternative to intraoral/extraoral wound suture because it is applied faster, prevents ischemia, and improves hemostasis. In general, a longer operative time is associated with a greater exposure to bacteria, larger amount of anesthetic, and higher morbidity rate [23]. In the present study, a significant reduction in operative time was achieved with the utilization of cyanoacrylate tissue adhesive (mean of 4.5 min) rather than suture (mean of 8 min). In the same line, Stavropoulou et al. [14] reported that the operative time was threefold shorter with cyanoacrylate than with conventional suture. In the setting of maxillofacial surgery, Soni et al. [24] found that the time saved by using cyanoacrylate increased with a greater incision length, which requires more sutures, but not a longer cyanoacrylate application.

In both groups of patients, pain was most intense during the first 48 h post-surgery and then progressively decreased until it disappeared, in agreement with previous studies of this type [3]. In the present study, the variation in pain over the first 7 days was much wider in the suture group owing to its higher initial intensity in comparison with the cyanoacrylate tissue adhesive group. Tavelli et al. [25] also attributed a more abrupt decrease in pain in the control group during the first 48 h to its greater initial intensity.

No significant between-group differences were found in postoperative pain, as also reported by Zucchelli et al. [21], who evaluated the pain according to the need for analgesic medication, and by Stavropoulou et al. [14], who closed the graft by primary intention. In a larger sample of patients (n = 60 in each group), Oladega et al. [26] also found no between-group difference in the patients’ experience of pain. A significant difference in pain was described by Ozcan et al. [6] between the utilization of platelet-rich fibrin with cyanoacrylate tissue adhesive and the absence of any wound closure material. A significant improvement in post-operative pain was reported using a cyanoacrylate-treated collagen sponge compared with suture [25] or with a cyanoacrylate-free collagen sponge [7]. In general, researchers have described pain as being most intense during the first 48 h and significantly lesser with the application of cyanoacrylate tissue adhesive.

A significant between-group difference was found in spontaneous bleeding of the surgical wound during the first 48 h. Ozcan et al. [6] and Oladega et al. [26] also described a statistically significant difference in postoperative bleeding during the first day post-surgery. However, no difference in bleeding was observed by Stavropoulou et al. [14] or by Griffin et al. [27], who attributed the bleeding more to trauma produced during the postoperative period than to possible deficiencies of the technique, as also argued by Escobar et al. [28]. This discrepancy with the present results may be explained by possible traumas during the post-surgical period, which are more frequent when the bleeding area is not covered by a rigid layer, as is the case when cyanoacrylate adhesive is used. In the present study, cases of postsurgical hemorrhage refer to small amounts of bleeding that do not need emergency treatment, only compression for 30 min with gauze soaked in physiological serum.

The timings of first epithelial layer formation and restitutio ad integrum were evaluated [29], detecting this epithelial layer in virtually all patients at 21 days post-surgery. No between-group difference was observed, as also reported by Stavropoulou et al. [14]. However, other authors found a significantly shorter healing time in patients treated with cyanoacrylate [30]. Some patients in both of the present groups showed early epithelial formation at 15 days, as also observed by Vastani and María [9], although they harvested smaller grafts. Notwithstanding, other authors have highlighted a faster healing using cyanoacrylate owing to the fact that the adhesive acts as a scab [31], where the keratinocytes play a pivotal role [32]. Zucchelli et al. [2] concluded that the healing time was influenced by the size of the bloody area, which was not taken into account in the present study. Complete healing was observed in all patients in both groups at two months.

The main study limitation was the small sample size, although it was adequate to reveal statistically significant differences in operative time and in the presence of spontaneous bleeding during the first day post-surgery. It is possible that differences observed in other variables (e.g., pain) might have reached statistical significance with a larger sample size. A further limitation was that no data were gathered on the width or surface area of the surgical wound, which has previously been reported to influence pain outcomes and wound healing time [18]. On the other hand, the size of grafts would have been similar in all of the present patients, given that they were harvested to cover gingival recession defects in anterior teeth. Furthermore, other authors [2,7] concluded that the postoperative pain was more strongly influenced by measures to protect the bloody area than by the graft size. However, the size of the graft, especially the horizontal dimension, may play a role in wound healing [33], and the earlier formation of the first epithelium layer (at 15 days) in some patients may be attributable to a shorter transversal distance to the bloody area in comparison with the other patients.

## 5. Conclusions

The utilization of cyanoacrylate tissue adhesive rather than suture to close the wound in the palatal donor area after epithelial-connective tissue graft harvesting reduces the operative time and the bleeding during the first 24 h post-surgery.

## Figures and Tables

**Table 1:** Pain VAS score during the first 14 days.

	Suture (n = 14)			Cyanoacrylate Tissue Adhesive (n = 10)			
Pain	Median	Mean	SD	Median	Mean	SD	*p*-Value *
Day 1	3.00	4.21	2.89	3.50	3.20	1.87	0.625
Day 2	2.00	2.79	2.29	3.00	2.30	1.89	0.841
Day 3	1.50	2.14	1.99	2.00	1.90	1.97	0.796
Day 4	2.00	2.00	1.62	2.50	2.10	2.08	0.977
Day 5	2.50	2.14	1.66	2.00	1.80	2.39	0.312
Day 6	1.50	2.21	2.19	0.50	1.30	2.45	0.122
Day 7	1.00	1.29	1.33	0.00	1.10	2.47	0.259
Day 14	0.00	0.50	0.94	0.00	0.70	2.21	0.546

* *p*-value by Mann–Whitney U test.

**Table 2:** VRS-evaluated inflammation at 7, 14, and 21 days.

	Suture (n = 14)				Cyanocrylate Tissue Adhesive (n = 10)				
	None	Mild	Marked	Extreme	None	Mild	Marked	Extreme	*p*-Value *
7 days	0%	28.6%	71%	0%	0%	50%	50%	0%	0.403
14 days	14.3%	57.1%	28.6%	0%	10%	80%	10%	0%	0.470
21 days	57.1%	42.9%	0%	0%	70%	30%	0%	0%	0.678

* *p*-value by Mann–Whitney U test.

**Table 3:** Bleeding at 24, 48, and 72 h.

	Suture (n = 14)		Cyanoacrylate Tissue Adhesive (n = 10)		
Bleeding	NO	YES	NO	YES	*p*-Value *
24 h	42.9%	57.1%	90%	10%	0.033
48 h	78.6%	21%	100%	0%	0.239
72 h	85.7%	14.3%	100%	0%	0.493

* *p*-value by Fisher’s exact test.

**Table 4:** Healing at 14 and 21 days.

	Suture (n = 14)	Cyanoacrylate Tissue Adhesive (n = 10)	
Epithelium Formation	%	%	*p*-Value *
14 days	21.4%	30%	0.665
21 days	78.6%	70%	0.665

* *p*-value computed by Fisher’s exact test.

## Data Availability

The data presented in this study are available on request from the corresponding author.
